# SwapMyMood: User-Centered Design and Development of a Mobile App to Support Executive Function

**DOI:** 10.1007/978-3-030-58805-2_31

**Published:** 2020-08-12

**Authors:** Tracey D. Wallace, John T. Morris

**Affiliations:** 8 Institute Integriert Studieren, JKU Linz, Linz, Austria; 9 Jack Baskin School of Engineering, UC Santa Cruz, Santa Cruz, CA USA; 10 Dipartimento di Meccanica, Politecnico di Milano, Milan, Italy; 11 Support Centre for Students with Special Needs, Masaryk University Brno, Brno, Czech Republic; Crawford Research Institute, Shepherd Center, Atlanta, GA 30309 USA

**Keywords:** eHealth, Brain injury, Executive function, Problem solving, Emotion regulation

## Abstract

This paper describes the research and development of the SwapMyMood smartphone application designed to support use of evidence-based executive function strategies by people with traumatic brain injury. Executive dysfunction is a common sequela of traumatic brain injury (TBI) resulting in diminished cognitive-behavioral functioning. Problem-solving and emotion regulation are cognitive-behavioral functions that are often disrupted by changes in the executive control system. SwapMyMood is an electronic version of the Executive Plus/STEP program, a set of clinical techniques taught to people living with brain injury to help them 1) identify and implement solutions to problems encountered in daily life and 2) to utilize the emotion cycle to understand and regulate emotional responses to these problems. The Executive Plus/STEP program has until now relied on paper-based instruction and use. Input from target users – people with brain injury and clinical professionals who teach this program to their patients – has contributed to key refinements of features and functioning of the mobile app. Data gathered from target user participation in the user-centered design process are presented. Future directions for ongoing development of technologies to support executive function strategies are also discussed.

## Introduction

The paper describes the research and development of the SwapMyMood smartphone application designed to support use of evidence-based executive function strategies by people with traumatic brain injury (TBI). SwapMyMood is an electronic version of the Executive Plus/STEP program, a set of clinical techniques taught to people living with brain injury to help them 1) identify and implement solutions to problems encountered in daily life and 2) utilize the emotion cycle to understand and regulate their emotional responses to these problems. Data gathered from target users who participated in the design process are presented. Future directions for ongoing development of technologies to support executive function strategies are also discussed.

Executive dysfunction is a common sequela of traumatic brain injury (TBI) resulting in diminished cognitive-behavioral functioning. Problem-solving and emotion regulation are cognitive-behavioral functions that are often disrupted by changes in the executive control system. Such changes can impact a person’s independence community integration. Cognitive rehabilitation literature supports systematically training metacognitive strategies—particularly problem-solving strategies—to support executive functioning in people with TBI [1–4]. The literature supports use of a comprehensive, formal model of problem-solving [3–6].

The Executive Plus/STEP program is an evidence-based model incorporating instruction in metacognitive strategy and training in formal problem-solving to support problem-solving (using SWAPS strategy) and emotion regulation (using Emotion Cycle strategy). An RCT of the Executive Plus/STEP program concluded it is efficacious in improving self-reported post-TBI executive function and problem solving [7].

## State of the Art

The Executive Plus/STEP program was adapted for use in Shepherd Center’s SHARE Military Initiative, a comprehensive rehabilitation program focused on assessment and treatment of service members with TBI and Post-Traumatic Stress Disorder (PTSD). Data gathered using the National Outcome Measurement System (NOMS) created by the American Speech-Language-Hearing Association (ASHA) were used to evaluate program outcomes following implementation of this adapted approach within the SHARE program. Program outcomes data were analyzed for 95 clients with chronic symptoms of TBI who received this problem-solving intervention, revealing 86% improved by at least one level on the Adult NOMS Problem Solving Functional Communication Measure and 54% improved by two or more levels.

Despite successful adaptation and implementation of the Executive Plus/STEP program, many SHARE clients report difficulty recalling the multiple steps in the interventions, recalling which strategies they have used successfully and initiating strategy use under stress. When learning the strategies, clients routinely refer to a written workbook that describes the Executive Plus/STEP program interventions. But they often do not have the workbook with them for support when problems arise.

We developed the SwapMyMood mobile app for iOS to make the Executive Plus/STEP program more accessible and useful wherever the user may be. SwapMyMood is designed to assist people with TBI in using evidence-based methods to maximize problem-solving and emotion regulation. The goal was to make it portable while including functionality enabled by the electronic interface and cloud-based storage/interaction. Development of a smartphone app makes information contained in the workbook more accessible and portable by providing an easily navigable electronic version of these tools which can be carried in the user’s pocket. The electronic version also has the potential to provide users with added support for completing each step in the intervention and recalling previous strategies used, which can be saved and retrieved quickly.

## Research and Development of SwapMyMood

Development of SwapMyMood employed user-centered design principles by including target users throughout the design process [8, 9]. The initial design concept was led by a speech-language pathologist with expertise in TBI cognitive rehabilitation and a psychologist with expertise in TBI/PTSD.

Seven SMEs (3 speech pathologists, 3 Clinical Psychologists/Social Workers and 1 author or the Executive Plus/STEPS Manual) and 6 target users with TBI contributed to the development via interviews, sit-by demonstrations and take-home testing. Interviews and sit-by demonstrations lasted approximately 60 min. Take-home testing lasted 2 weeks.

Participants with TBI were recruited from the SHARE Military Initiative at Shepherd Center. Subject matter experts were recruited from the researchers’ professional networks. The inclusion criteria of participants with TBI are listed below:

- Traumatic brain injury – Greater than 2 weeks post-onset.
- Identified by their speech-language pathologist and behavioral health provider as an appropriate candidate to use the SWAPS and Emotion Cycle interventions.
- 18 years of age, or older.
- Functional hearing & vision.
- Ability to follow 2 step directions (English speaking).
- Functional reading.

Inclusion criteria for the Subject Matter Experts (SME) were: 1) licensed clinician in the United States, and 2) experience using the SWAPS and Emotion Cycle interventions with people with TBI.

## Results

The Executive Plus/STEP program is complex with a numerous actions that require users to input information related to problems they are facing and aspects of the emotion cycle the observe in their current state. Translating the substantial training manual into an app requires considerable effort to ensure intelligibility of the overall flow and perceivability of the information presented.

Feedback from interviews and sit-by demonstrations informed solution features, including:

- Guidance through the multiple steps of problem-solving and emotion regulation.
- Inclusion of video tutorials.
- User selected customization options.
- Links to strategy banks and useful tips based on the Executive Plus/STEP manual.
- Functionality to record, edit and save information input for future reference.
- Functionality to send strategy plans to a caregiver via email.

Seven SMEs and 4 target users with TBI participated in sit-by demonstrations. Most gave positive feedback:

- “It looks great.”
- “Really well-designed.”
- “That’s really cool.”
- “I like it. It’s not as clunky as I thought it was going to be.”

Target users with TBI identified specific features or qualities of SwapMyMood that were strongly appreciated and those that were cause for concern (Table 1). They liked the accessibility of the digital format compared to the paper-based manual, and they like other features like recall and prompting of the user with information previously entered and the phone-a-friend feature. User concerns focused on the complexity like the inability to view all the text on some screens and the number of dropdown menus and options within those menus. The original version of the app that served as the basis for testing also had a limited color palette, which was also not perceived as ideal. Enhancements made to the app based on feedback include:

**Table 1.** Summary of feedback from sit-by target users.

Most favored features	Concerns
Accessibility of digital mobile format	Can’t see all text when phone is vertical
Strategy prompting & recall of entered information	Too many dropdown boxes
Phone a friend is “crucial”	Need more color schemes
Ability to review past strategy plans	Need to be able to edit entries
Using app during stress is “grounding”

- Design changed to permit optimal viewing when screen is in vertical position.
- Reduction of dropdown boxes.
- Ability to edit and email strategy plans.

As part of our user-centered design process, we invited two target users with TBI to use the app in their daily lives for two weeks to allow the user to explore the solution more fully and under more varied conditions. Results show greater knowledge and use of the problem solving and emotion regulation techniques in the Executive Plus/STEP program by Target User 1 (Table 2).

**Table 2.** Summary of feedback from take-home target user 1.

	Before testing app	After testing app
How well do you know use of SWAPS	I know it a little	I know it well
How well do you know use of the emotion cycle?	I know it a little	I know it well
How often do you use your SWAPS and Emotion Regulation workbooks/worksheets (asked before) or the app (asked after)?	Less than half the times when I use the strategies	Most of the times when I use the strategies

Target User 2 reported no change in the levels of knowledge and use over the testing period: high levels of knowledge of problem solving and emotion regulation techniques and moderate levels of use of these techniques (Table 3).

**Table 3.** Summary of feedback from take-home target user 2.

	Before testing app	After testing app
How well do you know use of SWAPS	I know it well	I know it well
How well do you know use of the emotion cycle?	I know it well	I know it well
How often do you use your SWAPS and Emotion Regulation workbooks/worksheets (asked before) or the app (asked after)?	Less than half the times when I use the strategies	Less than half the times when I use the strategies

## Discussion: Scientific and Practical Contribution to the Field

User-centered design resulted in several iterations of the SwapMyMood app and continues to inform work on major version updates currently underway. Initial interviews, sit-by testing sessions and take-home testing supported the general concept of developing an app-based version of the Executive Plus/STEP intervention. Notably, the take home testing participant who expressed little confidence in his ability to use the strategies reported a marked increase in confidence after using the app for 2 weeks. He also reported using the app for support more frequently than he used the paper workbook prior to using the app.

Informed by a user-centered design approach, the SwapMyMood mobile app extends findings from other research on problem solving and executive functioning by people with brain injury. Rath, Hennessy and Diller (2003) found that social problem solving (SPS) is an important component of community integration following traumatic brain injury (TBI). These researchers stressed the importance of assessing a persons’ confidence in their ability to cope with problems after brain injury; a focus on objective test scores alone may lead to under-detection of disabling problem-solving deficits. SwapMyMood provides a portable set of potentially useful tools and allows the user and clinician to track historical problem-solving performance in daily life.

Other researchers have developed and tested of an electronic version of an intervention to support emotion regulation or problem solving, but not both. Ehlhardt, et al. report on development and evaluation of a web-based program to support problem-solving skills after brain injury [10]. Other evidence-based electronic solutions include BEST Connections suite of mobile apps for people with cognitive limitations including those caused by brain injury. The suite includes 4 discrete but integrated solutions for planning and self-management, not for problem solving and emotion regulation. These solutions, however, point to the utility of electronic supports for people with brain injury and other conditions that cause executive function challenges.

## Conclusion and Future Directions

Additional development of the app is currently underway. Version 2.0 will be released on Android and iOS in June 2020. It will include enhanced navigation, including the ability to pause/resume the problem-solving process in order to complete the emotion cycle to regulate emotions which may be interfering with effective problem solving. The new version also will improve display of summaries of each problem-solving instance and retrieval of previous problem-solving instances. The visual design of the interface – logo, colors, general layout of each screen – will be enhanced, as well.

Launch of SwapMyMood 2.0 will allow testing for clinical efficacy. The team will conduct a pragmatic clinical trial to compare outcomes in people with TBI who use SwapMyMood versus the paper workbook. Improvement in executive functioning, frequency of app use, types of problems encountered, strategies selected and satisfaction with outcomes of strategy use will be assessed. Study design will be a between-subjects test with random assignment to two groups of clients in Shepherd Center’s SHARE military and veterans rehabilitation: 1) those receiving conventional training in the Executive Plus/STEP program and use of the workbook only; and 2) those receiving conventional training with the workbook and the SwapMyMood mobile app, with subsequent use of the SwapMyMood app only. Participants in both groups will utilize the Executive Plus/STEP program in their respective formats for 1 month. Participant use of each format will be recorded in weekly conversations with their speech therapist during treatment sessions. Additionally, use of the mobile app will be recorded in the app’s Amazon Web Services (AWS) cloud-based reporting system.

The R&D team is already planning version 3.0 which will incorporate machine learning (ML) and artificial intelligence (AI) to support context awareness and predictive modeling to anticipate the user’s need to perform either or both problem-solving and the emotion cycle. The team expects that these features and functions will enhance support for the user by delivering timely prompts, reminders and engagement, while minimizing risk of over-communicating with the user.
